# Uppermost Triassic phosphorites from Williston Lake, Canada: link to fluctuating euxinic-anoxic conditions in northeastern Panthalassa before the end-Triassic mass extinction

**DOI:** 10.1038/s41598-019-55162-2

**Published:** 2019-12-11

**Authors:** Ekaterina Larina, David J. Bottjer, Frank A. Corsetti, John-Paul Zonneveld, Aaron J. Celestian, Jake V. Bailey

**Affiliations:** 1 Earth Sciences, University of Southern California, Los Angeles, USA; 2 Earth and Atmospheric Sciences, University of Alberta, Edmonton, Canada; 3 Mineral Sciences, Natural History Museum, Los Angeles, USA; 4 Earth Sciences, University of Minnesota, Minneapolis, USA

**Keywords:** Climate change, Palaeoceanography

## Abstract

The end-Triassic mass extinction (ETE) is associated with a rise in CO_2_ due to eruptions of the Central Atlantic Magmatic Province (CAMP), and had a particularly dramatic effect on the Modern Fauna, so an understanding of the conditions that led to the ETE has relevance to current rising CO_2_ levels. Here, we report multiple phosphorite deposits in strata that immediately precede the ETE at Williston Lake, Canada, which allow the paleoenvironmental conditions leading up to the mass extinction to be investigated. The predominance of phosphatic coated grains within phoshorites indicates reworking in shallow water environments. Raman spectroscopy reveals that the phosphorites contain organic carbon, and petrographic and scanning electron microscopic analyses reveal that the phosphorites contain putative microfossils, potentially suggesting microbial involvement in a direct or indirect way. Thus, we favor a mechanism of phosphogenesis that involves microbial polyphosphate metabolism in which phosphatic deposits typically form at the interface of euxinic/anoxic and oxic conditions. When combined with data from deeper water deposits (Kennecott Point) far to the southwest, it would appear a very broad area of northeastern Panthalassa experienced anoxic to euxinic bottom water conditions in the direct lead up to the end-Triassic mass extinction. Such a scenario implies expansion and shallowing of the oxygen minimum zone across a very broad area of northeastern Panthalassa, which potentially created a stressful environment for benthic metazoan communities. Studies of the pre-extinction interval from different sites across the globe are required to resolve the chronology and spatial distribution of processes that governed before the major environmental collapse that caused the ETE. Results from this study demonstrate that fluctuating anoxic and euxinic conditions could have been potentially responsible for reduced ecosystem stability before the onset of CAMP volcanism, at least at the regional scale.

## Introduction

**Figure 1:**
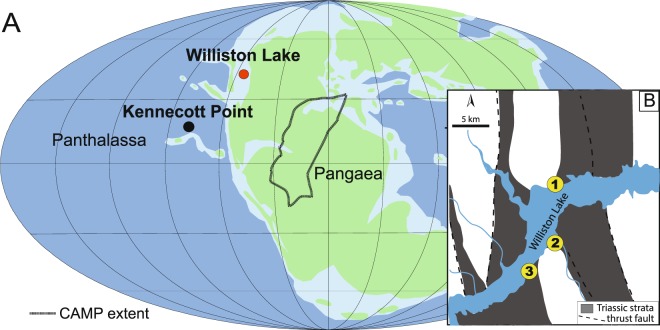
Paleogeography and modern location of study localities. (**a**) Early Jurassic paleogeographic reconstruction with the approximate paleolocation of Williston Lake sites after Greene *et al*.^14^ and Kennecott Point site after Kasprak *et al*.^18^, and outlined area within Pangaea showing the extent of CAMP. (**b)** Map of the studied localities at Williston Lake, British Columbia modified from Wignall *et al*.^24^ 1 = Black Bear Ridge (56.08758333°N, 123.04388889°W), 2 = Pardonet Creek (56.03527778°N, 123.03500000°W), 3 = Ne Parle Pas (56.01682778°N, 123.08305556°W).

**Figure 2:**
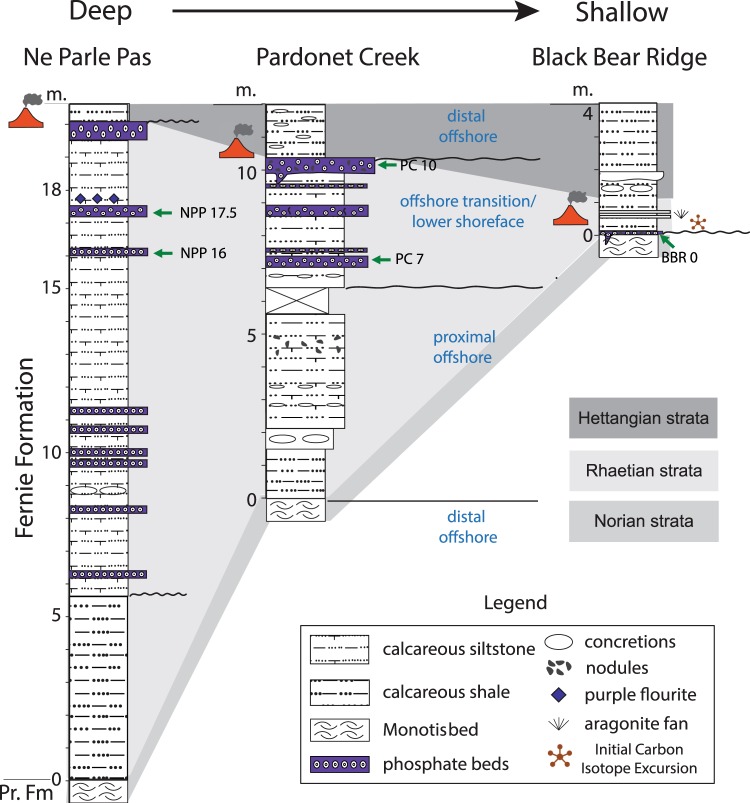
Stratigraphic columns of studied sections showing depositional environment. Green arrows show the position of studied samples depicted in the figures below. Placement of Initial Carbon Isotope Excursion is after Wignall *et al*.^24^. Volcano marks the end-Triassic mass extinction. Aragonite fans described in Greene *et al*.^15^ Pr. Fm = Pardonet Formation.

The end-Triassic mass extinction (ETE) appears nearly coincident with the emplacement of the Central Atlantic Magmatic Province (CAMP) ~201.51 million years ago^1–5^. Approximately 80% of all marine and terrestrial species became extinct during the ETE making it the second biggest biodiversity^6,7^ and the third biggest ecological crisis^8^ during the Phanerozoic. Proposed causes of the mass extinction include CAMP-related global warming^4,9,10^, global cooling^11^, ocean acidification^12–15^, sea-level changes^16^, and ocean anoxia^3,16–18^. The knowledge base of the conditions leading up to the ETE is poorly resolved, as the main focus has remained on the timing associated with an initial emplacement of CAMP. Yet, recent studies of the pre-extinction interval reveal biotic, climatic and geochemical changes before the onset of known CAMP volcanism^19–22^. Here, we present the first detailed study investigating the genesis of Upper Triassic phosphorite deposits from Williston Lake, British Columbia, which intriguingly occur directly before the ETE interval (Figs. 1 and 2).

Phosphorite beds at Williston Lake are predominantly composed of phosphatic coated grains. Deposits containing phosphatic coated grains are rare throughout the Phanerozoic and their origin remains incompletely understood^23–25^. It is widely accepted that direct precipitation of phosphates occurs near the sediment-water interface and requires distinct environmental conditions^26–29^. Formation of authigenic phosphorites (phosphogenesis) is generally associated with microbial polyphosphate metabolism and biogeochemical processes where steep, or temporally dynamic, redox gradient conditions occur^26–28^. Pufahl and Grimm^29^ suggested that phosphatic coated grains are proxies for fluctuations in organic carbon flux and primary productivity. Our study suggests that the formation of these phosphatic coated grain deposits was microbially mediated based on textural features and Raman spectroscopy. While phosphorites are an indicator of steep or dynamically-variable redox gradient conditions, the occurrence of benthic macrofauna within phosphatic beds implies that transient oxygenation events occurred during phosphogenesis^24^. Our results demonstrate a development of oxygen restricted conditions, which would be stressful to most benthic metazoans established in shelf waters off northeastern Panthalassa prior to the ETE and preceding the earliest known CAMP volcanism.

### Study area

Exposures of the Fernie Formation along Williston Lake, British Columbia, Canada span the Triassic/Jurassic boundary and include the record of the entire Rhaetian Stage, the final stage of the Triassic across different environments^24^ (Fig. 2). The siliciclastic-carbonate strata were deposited in a foreland basin on the western side of the Pangaean continental shelf ^30,31^ (Fig. 1). The Rhaetian Stage is marked by sea-level fall followed by a major transgression at the end of the Rhaetian coincident with an initial worldwide negative carbon isotope excursion^24,31^(Fig. 2).

We investigated three sections (Ne Parle Pas Point, Pardonet Creek, and Black Bear Ridge) that contain phosphorite deposits in the interval immediately preceding the ETE. The sea-level fall during the Rhaetian generated a major hiatus in the more proximal Black Bear Ridge section such that nearly the entire Rhaetian sequence is missing at this locality; nevertheless, a veneer of phosphatic coated grains is preserved at the sequence boundary. Strata recording a more complete Rhaetian Stage are at the distal sites of Ne Parle Pas Point and Pardonet Creek where both sites record lowstand strata missing at the Black Bear Ridge locality (Fig. 2). The upper 13 m of the Ne Parle Pas section and the upper 3.5 m of the Pardonet Creek sequence consist of calcareous siltstone interbedded with phosphatic grainstone beds recording a transition from offshore to lower shoreface depositional environments (Fig. 2). In general, phosphorite accumulations are associated with a condensed, transgressive record^32^, yet intriguingly Williston Lake phosphorites are deposited within the regressive sequence^24^ (Fig. 2).

## Results

### Description of phosphorite deposits

**Figure 3:**
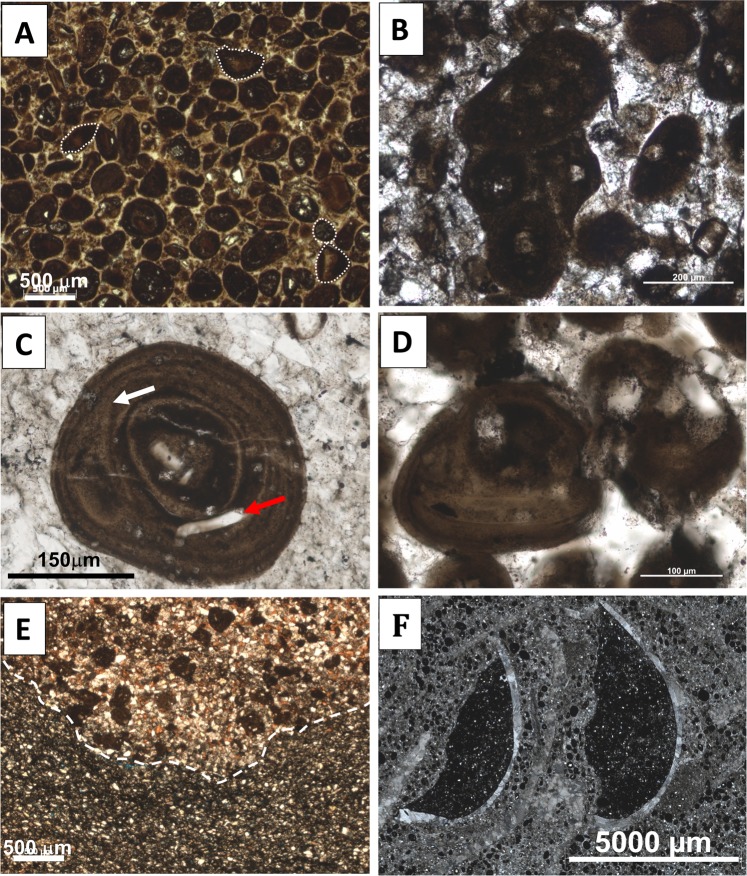
Photomicrographs of phosphatic coated grains (PCG). All images are in plane-polarized light. (**a)** Fragmented phosphatic coated grains. Note size and shape variation. Sample PC 10. (**b)** Phosphatic coated grains embedded within phosphatic crust resembling grapestone structure with phosphatic peloids. Sample NPP17.5. (**c)** Light and dark brown concentric laminations. White arrow points to the divergence area where outer laminae replicate shape of a clast within cortex. Red arrow points to unaltered shell fragment within the cortex of phosphatic coated grain. Sample PC 7. (**d)** Phosphatic coated grain with irregular concentric laminations. Sample NPP 17.5. (**e)** Coarser grained P-rich sediment separated by scouring surface from underlying finer-grained sediment. Sample NPP 16. (**f)** Bivalve clasts filled with silt and peloids that were ripped up and transported by storms. PC7

The phosphorite beds studied here represent high-energy phosphatic grainstone beds that vary from 5 to 40 cm in thickness (Fig. 2). The grainstone beds represent storm events sandwiched between calcareous siltstone to sandstone deposits. Phosphatic clasts were transported from a shallower part of the shelf to the deeper offshore transition zone during high-energy, storm-related events as evidenced by fragmented grains, rip-ups, phosphate grapestones and scouring surfaces between fine-grained siltstone and coarse-grained phosphatic grainstone beds (Fig. 3A,B,E,F). The phosphorite deposits predominantly consist of phosphatic coated grains with some peloids, muscovite, quartz silt, bioclasts and phosphatic pebbles embedded in a sparry calcite matrix. According to Wignall *et al*.^24^, the phosphatic units contain some of the more biotically diverse assemblages versus surrounding units, and include infaunal and epifaunal bivalves, nautiloids, ammonoids, and echinoderms. In all localities, phosphatic coated grains vary in shape from spheroids to ellipsoids and are predominantly fragmented due to reworking and winnowing (Fig. 3A). Occasionally phosphatic coated grains embedded in a phosphatic matrix are present (Fig. 3B). Phosphatic coated grains are 50 to 380 μm in diameter, more commonly between 100 and 150 μm. Phosphatic cortices are uniformly composed of Ca-phosphate (apatite) while nuclei composition includes sedimentary clasts (most common), quartz, feldspar, and shell fragments. The nuclei of phosphatic coated grains are coated by concentric phosphatic laminae, which vary in thickness and alternate between light brown and dark brown layers (Fig. 3C,D). Concentric layers are irregular in places replicating the shape of the nucleus and occasional objects within laminae (Fig. 3C,D). The presence of concentric and irregular laminations along with unaltered bioclasts suggest the primary origin of phosphatic coated grains excluding the possibility of diagenetic replacement.

**Figure 4:**
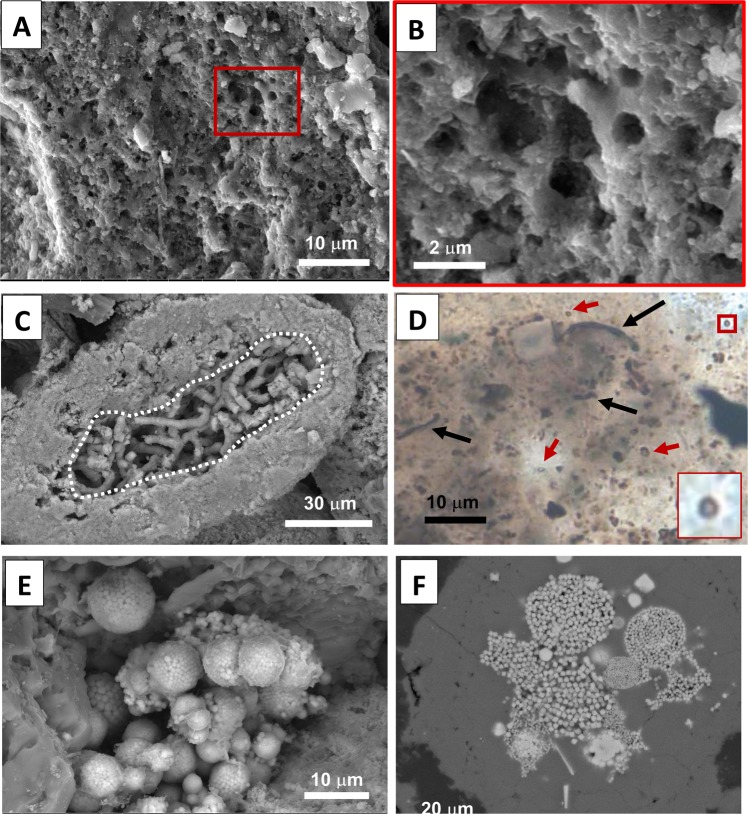
Photomicrographs and SEM images of putative microbial structures, euendolith borings, and pyrite framboids. (**a**) SEM image of microscopic spherical cavities (~0.5 – 1 μm in diameter) within PCG cortex. Sample BBR0. (**b**) Close-up of image A. Sample BBR0. (**c**) SEM image of sample dissolved in acetic acid, which reveals phosphatized euendolith borings (dashed line). Sample NPP 17.5. (**d**) Photomicrograph in plane-polarized light of filamentous structures (black arrows) within phosphate and microscopical spherical cavities with distinct rim (red arrows) resembling microbial remains. Sample PC 7. (**e**) SEM image of sample dissolved in acetic acid, which reveals an aggregation of pyrite framboids. Sample NPP 17.5. (**f**) Backscatter SEM image of pyrite framboids within phosphate. Sample PC 7.

Samples dissolved in acetic acid from the Ne Parle Pas site that contain nuclei composed of calcium carbonate intraclasts reveal phosphatized tubes (Fig. 4C). Phosphatized tubes are between 2 and 3 microns in diameter creating an interwoven network identical in shape, size, and structure to endolithic borings^33^. Thus, these carbonate intraclasts were infested by euendoliths during “normal” conditions followed by rapid phosphatization before micritization could occur.

### Microbial mineralization

**Figure 5:**
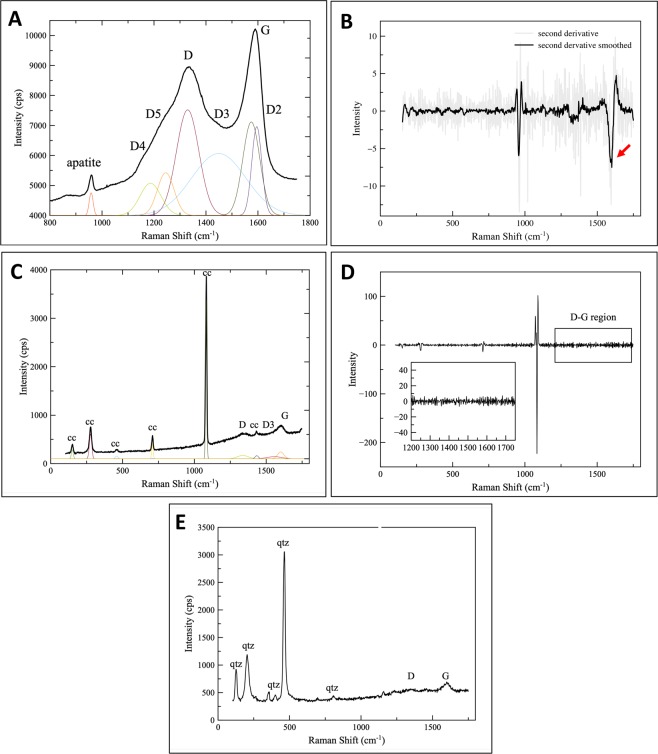
Raman spectroscopic analysis. (**a**) Representative Raman spectrum of phosphate cortex showing high intensity peaks of D-G bands and deconvolution of D and G bands modeled by six Gaussian peaks below (fitted peaks are scaled for clarity). High intensity peaks of D-G bands and big difference between bands D4 and D verify the biogenic origin of carbonaceous matter^34^. Sample PC7. (**b**) The second derivative spectra derived from the first order carbon spectra (**A**) shows no negative doublet on the negative peak at the G band (see red arrow) corroborating the presence of biogenic carbonaceous material within phosphate cortices. Sample PC7. (**c**) Representative Raman spectrum of calcareous matrix showing low intensity peaks of D-G bands and deconvolution of D and G bands modeled by three Gaussian peaks below (fitted peaks are scaled for clarity). CC = calcite. Sample PC7. (**d**) The second derivative spectra derived from the first order carbon spectra (**C**) shows absence of any intensity within D-G bands corroborating the absence of biogenic carbonaceous material within calcareous matrix. Sample PC7. (**e**) Representative Raman spectrum of nuclei of PCG showing low intensity peaks of D-G bands. QTZ = quartz. Sample PC7.

Raman spectroscopy is widely used as an analytical tool for differentiation between carbonaceous material of biogenic versus abiogenic origin in the rock record. At all Williston Lake localities, micro Raman spectroscopy documents intense and broad bands occurring at around 1350 cm^−1^ (D band) and 1600 cm^−1^ (G band) within phosphatic cortices which are diagnostic of organic matter (kerogen)^34^ (Fig. 5A). Further analysis of the deconvoluted carbon first-order spectrum derived from the D-G spectrum seen in Fig. 5A shows five bands resolved into Gaussian bands where the high difference between bands D and D4 can be observed (Fig. 5A). The second derivative spectra of the D and G bands illustrate that the G band doesn’t have a doublet on the negative peak (Fig. 5B). Both differences in D4 and D bands and the absence of a doublet on the negative peak of the G band corroborate the presence of disordered carbonaceous matter, likely of biological origin, within the phosphorites^34^. In contrast, Raman spectroscopic analysis conducted on the matrix and nuclei of phosphatic coated grains does not record features that support the presence of any kind of carbonaceous material (Fig. C, D).

SEM and petrographic examinations of studied samples show microscopic spherical cavities (0.5 to 1 μm in diameter) within phosphatic coated grains cortices (Fig. 4A,B,D). Photomicrographs of these cavities reveal a distinct rim and hollow core structure that rules out the possibility of abiotic precipitates that resemble microbial forms^35^. Cosmidis *et al*.^36^ documented coccus-like and rod-like biomorphs in a Miocene/Pliocene Peruvian phosphatic crust that are identical in shape, size and distribution to our microscopic spherical objects. Salama *et al*.^37^ showed that microspheres (0.5 to 2.5 μm in diameter) embedded in carbonaceous matrix from Upper Cretaceous phosphatic peloids are fossilized remains of coccoidal bacteria. Both studies concluded that the observed microspheres were once a part of microbial colonies that formed near the oxic-anoxic boundary in zones of sulfate reduction^36,37^. Thin section observations revealed the presence of curved filamentous structures, ~1 μm in width and varying in length from 5 to 27 μm (Fig. 4D), which we interpret as putative microbial remains. Observed microscopical spherical cavities and filamentous morphotypes within carbonaceous material indicate the bacterial origin of relatively diverse microbial communities.

The evidence for microbial mineralization is unsurprising as all of the currently accepted mechanisms of marine phosphogenesis require microbes to concentrate P either in a direct or an indirect way^36^. We would like to emphasize that the presence of microbes within the cortices of phosphatic coated grains do not necessarily imply their direct role in the apatite accretion within phosphatic coated grains, although such a scenario is plausible, but rather that they may have played a fundamental role in the delivery of P via polyphosphate metabolism. Documented pyrite framboids within phosphoclasts imply the presence of sulfide likely provided by sulfate reducing bacteria in reducing conditions^38^ (Fig. 4E,F). This is in accordance with the study by Wignall *et al*.^24^ which documented euxinic conditions based on framboidal size at Williston Lake sections.

## Discussion

Modern and ancient phosphorite deposits are mainly associated with oceanic upwelling regions associated with hypoxic oxygen-minimum zones (OMZ) overlying organic-rich sediments e.g.^27,28^. In such regions, phosphogenesis is thought to be driven by microbes that concentrate P as part of polyphosphate metabolism^27,28,38^, with the colorless sulfur bacteria, in addition to others, commonly considered key players. The sulfur bacteria live at interfaces between organic rich, sulfidic conditions below and oxic conditions above, and are thought to accumulate polyphosphate in order to help survive during prolonged, but fluctuating anoxic and sulfidic conditions^35^. Thus, many sedimentary phosphate concentrations indicate that environmental conditions cycled between oxic and euxinic/anoxic during phosphogenesis.

Mechanisms of authigenic apatite precipitation that do not involve polyphosphate metabolism include the sourcing of P from vertebrate bone^39^, or from dissolving Fe-oxide-bearing mineral phases, again under stratified redox conditions^27,40^. While these P sources are plausible drivers of Williston Lake phosphogenesis, we do not favor them as explanations. In the former case, fish bones are not abundant in the Williston Lake deposits and they do not serve as nuclei for the phosphatic coated grains. Similarly, with respect to P being concentrated through adsorption and subsequent dissolution of iron hydroxides, we would expect this mechanism to operate in a system dominated by iron-rich siliciclastic deposition, as opposed to the organic-rich system here that contains pyrite (Fig. 4E,F), indicating a sulfide-dominated depositional environment such as those that favor phosphogensis via concentration through microbial polyphosphate metabolism^41,42^.

Here, we consider the presence of phosphatic coated grains to indicate conditions fluctuated between oxic and sulfidic conditions in shallow water as a result of intensified oceanic upwelling and OMZ expansion in the lead up to the ETE in northeastern Panthalassa. The presence of phosphorites suggests that the foreland basin was well-connected to the ocean at the time of phosphogenesis. Models investigating genesis of phosphatic coated grains from Arning *et al*.^26^ and Pufahl and Grimm^29^ propose that phosphatic coated grains are deposited near the sediment-water interface (upper 5-20 cm of sediment) in organic-rich sediment under suboxic to anoxic conditions where repeated reworking of phosphatic grains and fluctuation of redox conditions play a crucial role. Although phosphatic coated grains are sometimes referred to as phospho-ooids, the process responsible for phospho-ooid formation is different from carbonate ooids, which precipitate by wave agitation in supersaturated waters with calcium carbonate^37^, while the apatite in phosphatic coated grains precipitates authigenically below the sediment-water interface^29^. We suggest that these phosphatic coated grains formed between storm and fair weather wave-base, but specifically in proximity to the wave-agitation zone where they were more prone to episodic reworking and mixing by storms and where the upwelling front would be best developed and intensified^32^. High-energy storm events reworked phosphate clasts further transporting them to the current place of deposition (phosphatic grainstone beds). The infilling of microbial borings in carbonate clasts by phosphate indicates relatively rapid phosphatization, before the borings were infilled by cements, or the microbial borers could fully micritize the carbonate grains (Fig. 4C).

The presence of putative microbial fossils within the phosphate cortices lends credence to a microbial involvement either in an indirect way by concentrating high amounts of P or in a direct way by facilitating an actual apatite accretion. The fact that the phosphatic beds contain macrofauna suggests a strong association with transient oxic conditions during which benthic metazoa colonized the seafloor until the next euxinic/anoxic event^24^. Taken together, the presence of phosphorites indicates a dynamic environment that shifted between oxic, hypoxic and possibly anoxic or euxinic conditions in the time preceding the ETE. Our study demonstrates evidence supporting microbial mineralization during the phosphogenesis either in a direct or an indirect way, likely by polyphosphate-metabolizing bacteria, similar to those that mediate phosphogenesis in modern settings that fluctuate between oxic and euxininc/anoxic conditions^25,38^.

Schoepfer *et al*.^20^ and Kasprak *et al*.^18^ investigated an open ocean, deep-water section (~200-500 m water depth) from northeastern Panthalassa, spanning the Triassic-Jurassic boundary several hundred km to the west of Williston Lake, at Kennecott Point, British Columbia (Fig. 1). Both of these studies interpret nitrogen-limited conditions, water-column stratification and deoxygenation at Kennecott Point through an ~10 m-thick interval, estimated to represent approximately half a million years^20^ before the ETE using geochemical and biomarker proxies. Although precise geochronology is problematic at Williston Lake, the timing of biogeochemical disturbance at Kennecott Point coincides well with deposition of the upper Rhaetian phosphate grainstone beds. Given that the Kennecott Point (deep) and Williston Lake (relatively shallow) sites are likely contemporaneous, it would appear that a broad area of northeastern Panthalassa experienced fluctuating euxinic-anoxic conditions in the lead up to the ETE, beginning ~500 kyr before the extinction. Fluctuating, oxygen-restricted conditions likely created a hostile environment for benthic fauna at least in shallow water settings where most benthos are less adapted to low oxygen conditions. For example, off the coast of Oregon, recent intensified upwelling brought oxygen-poor waters to shallow shelf depths (<50 m) where emergent anoxia nearly eradicated the macroscopic benthic invertebrate community and initiated the development of sulfide-oxidizing bacterial mats^43^. At Williston Lake sites, Wignall *et al*.^24^ documented the demise of shallow-water infaunal bivalves right before the initial negative carbon isotope excursion marking the major environmental collapse of the ETE. This stressed environment potentially made marine biota more susceptible to subsequent cascading effects from CAMP emplacement, at least in northeastern Panthalassa.

Photic zone euxinia combined with anoxic conditions is considered to be a potent mechanism for the ETE since these conditions are widely documented in Panthalassic and Tethys basins during the ETE^3,18^. It is worth noting that the first appearance of euxinia on the shelves, as suggested by the presence of upper Rhaetian phosphorites, could indeed precede the onset of CAMP volcanism, adding to the growing dataset that environmental conditions began to deteriorate before the beginning of CAMP volcanism in different parts of the globe^19,20,22^. Dike and sill intrusions in organic rich sediments in Brazil were proposed as a potential mechanism that drove the climate change preceding the onset of CAMP volcanism^19,21,44^. Study^44^ using pedogenic carbonate isotopes as a proxy for temperature variations has documented two periods of extreme temperature increase by 6 °C in congruence with atmospheric CO_2_ rise preceding the ETE. Thus, it is worth considering that intensified oceanic upwelling and OMZ expansion in northeastern Panthalassa was possibly related to climate warming as a result of higher *p*CO_2_. Additional studies are required to reveal conditions leading up the ETE which governed on the regional versus global scale.

## Conclusions

Spectroscopic, petrographic and SEM investigations on the genesis of uppermost Triassic phosphorites from Williston Lake, British Columbia, provide evidence supporting microbially-induced mineralization, likely by polyphosphate-accumulating bacteria. The genesis of phosphatic coated grains requires deposition near the sediment-water interface under suboxic to anoxic conditions where repeated reworking of phosphatic grains and fluctuation of redox conditions play an essential role^26,29^. Our study provides direct biosedimentary evidence that fluctuating euxinic-anoxic conditions existed in relatively shallow marine settings (above storm-wave base) preceding the ETE in northeastern Panthalassa. These results are compatible with previous studies of Schoepfter *et al*.^20^ and Kasprak *et al*.,^18^ from the deep-water Kennecott Point section, suggesting that environmental disturbances, likely episodic euxinia, preceded the ETE for approximately 500 kyr. For the first time, our study shows that euxinic-anoxic conditions existed not just in the deeper part of the northeastern Panthalassic basin prior to the ETE, but also in shallow water settings of the foreland basin covering a very broad area of northeastern Panthalassa. Intervals of phosphogenesis are linked to periods of an intensified oceanic upwelling and OMZ expansion where possibly sulfide-oxidizing microbial communities were thriving inducing apatite precipitation, while the benthic biota flourished in between euxinic/anoxic intervals, at least at the Williston Lake region. Such conditions were likely stressful for shallow water benthic metazoans making them more susceptible to cascading effects from CAMP volcanism. Additional studies from Panthalassic and Tethys basins are required to further elucidate conditions leading up to the ETE at the regional and global scale and their effects on ecosystems.

## Methods

Specimens from different phosphatic horizons covering all three studied sections were examined. Standard petrographic microscopy was performed on thin sections using a Zeiss Axio Imager.M2m equipped with a Zeiss HRc camera. Internal grain structure and elemental composition were investigated on a FEI Nova NanoSEM 450 in back-scattered electron mode with the help of an Energy-dispersive X-ray spectroscopy microprobe. To determine mineral content and the presence of kerogen, we used a Horiba XploRa PLUS Raman Microscope and Horiba XGT-7200 X-ray fluorescence microscope. Samples were dissolved in 10% acetic solution for 72 hours in order to observe the morphology of phosphatic clasts.

## Data Availability

All data generated or analysed during this study are included in this published article. All figures were created by authors of the paper.

## References

[1] Schaller MF, Wright JD, Kent DV, Olsen PE (2012). Rapid emplacement of the Central Atlantic Magmatic Province as a net sink for CO2. Earth and Planet. Sci. Lett.

[2] Blackburn TJ (2013). Zircon U-Pb geochronology links the end-Triassic extinction with the Central Atlantic Magmatic Province. Science.

[3] Jaraula CM (2013). Elevated pCO2 leading to Late Triassic extinction, persistent photic zone euxinia, and rising sea levels. Geology.

[4] Pálfy J, Kocsis AT (2014). Volcanism of the Central Atlantic magmatic province as the trigger of environmental and biotic changes around the Triassic–Jurassic boundary. Volcanism, Impacts and Mass Extinctions: Causes and Effects. Geol. Soc. of Am. Sp. Paper.

[5] Thibodeau AM (2016). Mercury anomalies and the timing of biotic recovery following the end-Triassic mass extinction. *Nature*. Communications.

[6] Sepkoski, J. Patterns of Phanerozoic extinction: a perspective from global data bases in Global events and event stratigraphy in the Phanerozoic. (ed. Walliser, O. H.) 35-51 (Springer, 1996).

[7] Alroy J. (2010). The Shifting Balance of Diversity Among Major Marine Animal Groups. Science.

[8] McGhee GR, Sheehan PM, Bottjer DJ, Droser ML (2004). Ecological ranking of Phanerozoic biodiversity crises: ecological and taxonomic severities are decoupled. Palaeogeogr. Palaeoclimatol., Palaeoecol..

[9] McElwain JC, Beerling DJ, Woodward FI (1999). Fossil plants and global warming at the Triassic-Jurassic boundary. Science.

[10] Wignall PB (2001). Large igneous provinces and mass extinctions. Earth Sci. Rev.

[11] Guex J, Bartolini A, Atudorei V, Taylor D (2004). High-resolution ammonite and carbon isotope stratigraphy across the Triassic–Jurassic boundary at New York Canyon (Nevada). Earth Planet. Sci. Lett..

[12] Hautmann M, Benton MJ, Tomasovych A (2008). Catastrophic ocean acidification at the Triassic-Jurassic boundary. N. Jb. Geol. Palaont. Abh.

[13] Kiessling W, Aberhan M, Brenneis B, Wagner PJ (2007). Extinction trajectories of benthic organisms across the Triassic–Jurassic boundary. Palaeogeogr. Palaeoclimatol. Palaeoecol.

[14] Greene SE (2012). Recognising ocean acidification in deep time: An evaluation of the evidence for acidification across the Triassic-Jurassic boundary. Earth Sci. Rev.

[15] Greene SE, Bottjer DJ, Corsetti FA, Berelson WM, Zonneveld JP (2012). A subseafloor carbonate factory across the Triassic-Jurassic transition. Geology.

[16] Hallam A (1981). A revised sea-level curve for the early Jurassic. Journal of the Geological Society.

[17] Hallam A, Wignall PB (2000). Facies changes across the Triassic–Jurassic boundary in Nevada, USA. J. Geol. Soc..

[18] Kasprak AH (2015). Episodic photic zone euxinia in the northeastern Panthalassic Ocean during the end-Triassic extinction. Geology.

[19] Ruhl M, Kürschner WM (2011). Multiple phases of carbon cycle disturbance from large igneous province formation at the Triassic-Jurassic transition. Geology.

[20] Schoepfer SD, Algeo TJ, Ward PD, Williford KH, Haggart JW (2016). Testing the limits in a greenhouse ocean: Did low nitrogen availability limit marine productivity during the end-Triassic mass extinction?. Earth Planet. Sci. Lett..

[21] Davies JH (2017). End-Triassic mass extinction started by intrusive CAMP activity. Nature communications.

[22] Yager JA (2017). Duration of and decoupling between carbon isotope excursions during the end-Triassic mass extinction and Central Atlantic Magmatic Province emplacement. Earth Planet. Sci. Lett..

[23] Piecha M (2002). A considerable hiatus at the Frasnian/Famennian boundary in the Rhenish shelf region of northwest Germany. Palaeogeogr. Palaeoclimatol. Palaeoecol.

[24] Wignall PB (2007). The end Triassic mass extinction record of Williston Lake, British Columbia. Palaeogr. Palaeoclimatol. Palaeoecol.

[25] Zoss, R. *et al*. Microbial communities associated with phosphogenic sediments and phosphoclast‐associated DNA of the Benguela upwelling system. *Geobiology*, 1–15 (2018). 10.1111/gbi.12318 30369004

[26] Arning ET (2009). Genesis of phosphorite crusts off Peru. Marine Geology.

[27] Crosby CH, Bailey J (2012). The role of microbes in the formation of modern and ancient phosphatic mineral deposits. Frontiers in microbiology.

[28] Hiatt EE, Pufahl PK, Edwards CT (2015). Sedimentary phosphate and associated fossil bacteria in a Paleoproterozoic tidal flat in the 1.85 Ga Michigamme Formation, Michigan, USA. Sedimentary Geology.

[29] Pufahl PK, Grimm KA (2003). Coated phosphate grains: Proxy for physical, chemical, and ecological changes in seawater. Geology.

[30] Orchard, M. J. *et al*. An intercalibrated biostratigraphy of the Upper Triassic of Black Bear Ridge, Williston Lake, northeast British Columbia. *Natural Resources Canada, Geological Survey of Canada*, (2001).

[31] Zonneveld JP, Beatty TW, Williford KH, Orchard MJ, McRoberts CA (2010). Stratigraphy and sedimentology of the lower Black Bear Ridge section, British Columbia: candidate for the base-Norian GSSP. Stratigraphy.

[32] Pufahl PK, Groat LA (2017). Sedimentary and igneous phosphate deposits: formation and exploration: an invited paper. Economic Geology.

[33] Tribollet A, Payri C (2001). Bioerosion of the coralline alga Hydrolithon onkodes by microborers in the coral reefs of Moorea, French Polynesia. Oceanologica acta.

[34] Marshall CP, Edwards HG, Jehlicka J (2010). Understanding the application of Raman spectroscopy to the detection of traces of life. Astrobiology.

[35] Mänd K (2018). Authigenesis of biomorphic apatite particles from modern upwelling zone sediments off Namibia. Geobiology.

[36] Cosmidis J, Benzerara K, Menguy N, Arning E (2013). Microscopy evidence of bacterial microfossils in phosphorite crusts of the Peruvian shelf: Implications for phosphogenesis mechanisms. Chemical Geology.

[37] Salama W, El-Kammar A, Saunders M, Morsy R, Kong C (2015). Microbial pathways and palaeoenvironmental conditions involved in the formation of phosphorite grains, Safaga District, Egypt. Sedimentary geology.

[38] Vietti LA, Bailey JV, Fox DL, Rogers RR (2015). 2015, Rapid formation of framboidal sulfides on bone surfaces from a simulated marine carcass fall. Palaios.

[39] Suess E (1981). Phosphate regeneration from sediments of the Peru continental margin by dissolution of fish debris. Geochim. Cosmochim. Acta.

[40] Ruttenberg KC, Berner RA (1993). Authigenic apatite formation and burial in sediments from non-upwelling continental margin environments. Geochim. Cosmochim. Acta.

[41] Goldhammer T, Brüchert V, Ferdelman TG, Zabel M (2010). Microbial sequestration of phosphorus in anoxic upwelling sediments. Nature Geoscience.

[42] Schulz HN, Schulz DH (2005). Large sulfur bacteria and the formation of phosphorite. Science.

[43] Chan F (2008). Emergence of anoxia in the California current large marine ecosystem. Science.

[44] Cleveland DM (2008). Pedogenic carbonate isotopes as evidence for extreme climatic events preceding the Triassic-Jurassic boundary: Implications for the biotic crisis?. Geological Society of America Bulletin.

